# Structural determinants in a glucose-containing lipopolysaccharide from *Mycobacterium tuberculosis* critical for inducing a subset of protective T cells

**DOI:** 10.1074/jbc.RA118.002582

**Published:** 2018-05-01

**Authors:** Prithwiraj De, Michael McNeil, Mei Xia, Claudia M. Boot, Danny C. Hesser, Karolien Denef, Christopher Rithner, Tyler Sours, Karen M. Dobos, Daniel Hoft, Delphi Chatterjee

**Affiliations:** From the ‡ Mycobacteria Research Laboratories, Department of Microbiology, Immunology and Pathology and; ¶ Central Instrument Facility, Department of Chemistry, Colorado State University, Fort Collins, Colorado 80523 and; § Department of Internal Medicine, Saint Louis University, St. Louis, Missouri 63104

**Keywords:** tuberculosis, vaccine, analytical chemistry, carbohydrate chemistry, carbohydrate function, γδ T cells, lipoglycan, structural analysis

## Abstract

Mycobacteria synthesize intracellular, 6-*O*-methylglucose–containing lipopolysaccharides (mGLPs) proposed to modulate bacterial fatty acid metabolism. Recently, it has been shown that *Mycobacterium tuberculosis* mGLP specifically induces a specific subset of protective γ_9_δ_2_ T cells. Mild base treatment, which removes all the base-labile groups, reduces the specific activity of mGLP required for induction of these T cells, suggesting that acylation of the saccharide moieties is required for γ_9_δ_2_ T-cell activation. On the basis of this premise, we used analytical LC/MS and NMR methods to identify and locate the acyl functions on the mGLP saccharides. We found that mGLP is heterogeneous with respect to acyl functions and contains acetyl, isobutyryl, succinyl, and octanoyl groups and that all acylations in mGLP, except for succinyl and octanoyl residues, reside on the glucosyl residues immediately following the terminal 3-*O*-methylglucose. Our analyses also indicated that the octanoyl residue resides at position 2 of an internal glucose toward the reducing end. LC/MS analysis of the residual product obtained by digesting the mGLP with pancreatic α-amylase revealed that the product is an oligosaccharide terminated by α-(1→4)–linked 6-*O*-methyl-d-glucosyl residues. This oligosaccharide retained none of the acyl groups, except for the octanoyl group, and was unable to induce protective γ_9_δ_2_ T cells. This observation confirmed that mGLP induces γ_9_δ_2_ T cells and indicated that the acylated glucosyl residues at the nonreducing terminus of mGLP are required for this activity.

## Introduction

*Mycobacterium* spp. produces many exotic lipids and glycolipids that have demanded exploration into their biological functions. Many of the glycans among these glycolipids are naturally methylated (1). Among these, two classes of methylated polysaccharides, 3-*O*-methyl mannopolysaccharide and 6-*O*-methylglucose–containing lipopolysaccharides (mGLPs)^2^ have been implicated in regulation of fatty acid synthesis (2–4). However, this has been contradicted by the observation that *Rv3032* and *MSMEG_5084* knockout mutants of *Mycobacterium tuberculosis* H37Rv and *Mycobacterium smegmatis*, known to be impaired in mGLP synthesis, displayed WT fatty acid contents (5). mGLPs have been found in several *Nocardia* species and in *Mycobacterium phlei*, *M. smegmatis*, *Mycobacterium bovis* bacillus Calmette-Guérin (BCG), *M. tuberculosis*, *Mycobacterium leprae*, and *Mycobacterium xenopi* (6–10). The characterization and biosynthesis of mycobacterial mGLP have been reported in detail over the last two decades (11–14). Current knowledge on biosynthesis of these methylated glucans suggested that acylation with acetyl, propionyl, isobutyryl, octanoyl, and succinyl groups from their respective acetyl-CoA and methylation occur simultaneously after the saccharide moiety has been assembled. Kamisango *et al*. (15) have proposed that mGLP is synthesized from the reducing end toward the nonreducing end through sequential glucosylation and methylation reactions.

The major function associated with mGLP in many previous reports has been fatty acid metabolism. However, an account of γ_9_δ_2_T cell–activating biological activity has been recently recognized (16). Several forms of mGLP were identified in the total mixture using ESI LC/MS: mGLP with one to three acetyls and an octanoyl; mGLP with isobutyryl, acetyls, and octanoyl; and mGLP with octanoyl, isobutyryl, three acetyls, and one succinyl residue. The location of the acyl functions was determined by 2D NMR and QTOF LC/tandem mass spectroscopy (MS/MS) experiments. Previous work has shown that in *M. phlei* the nonsuccinyl acyl substituents except octanoyl are on the terminal 3-*O*-Me Glc and with some evidence that it may also have two acyl groups at 4 and 6 positions (6).

In this work, we have shown that a C_18_ reverse phase–based nanoLC-nanoESI-MS/MS analysis of methylated acylated glycans in negative ion mode is possible and that diagnostic fragment ions can allow determination of the location of the acyl functions with some confidence. Although these molecules have complex structures and molecular weights that range from 3500 to 4000, the spectra are completely interpretable and are consistent with the previous structural assignments. In addition, they reveal subtle features that were not apparent from earlier studies (6, 17) and contribute to novel biological activity (16).

## Results

### ^1^H PRESAT NMR of deacylated mGLP (mGP) and its effect on γ_9_δ_2_ T cells

**Figure 1.**
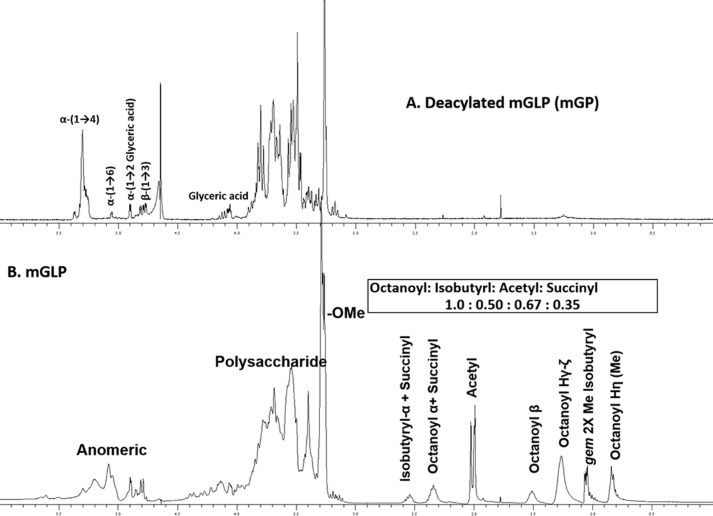
**^1^H NMR.** *A*, mGP. The deacylation was achieved by mild base hydrolysis followed by desalting. The anomeric region (δ 4.7–5.5 ppm) revealed the type of glycosidic linkages present in mGLP backbone. *B*, mGLP. The aliphatic region (between δ 0.5 and 3.0 ppm) revealed the different acyl groups present in mGLP. The succinyl group was confirmed with HSQC NMR (in Fig. 2).

Purified mGLP was treated with mild base, and the resulting product was desalted using a Bio-Gel (P-2) column and examined by NMR (Fig. 1*A*) (4). The resulting mGP revealed well-resolved anomeric protons in ^1^H NMR. A clear doublet at δ 4.89 ppm (*^3^J* = 3.76 Hz) was attributed to the anomeric proton of α-Glc*p*-(1→2)-glyceric acid. The two overlapping doublets at δ 4.77 (^3^*J* = 7.9 Hz) and δ 4.78 ppm (^3^*J* = 8.2 Hz) confirmed the presence of two β-Glc*p*-(1→3)-Glc*p*–linked residues. The presence of one α-Glc*p*-(1→6)-α-Glc*p* linkage was evident at δ 5.05 ppm (^3^*J* = 3.84 Hz). The overlapping cluster of peaks between δ 5.25 and δ 5.38 integrated to 16 protons and was assigned to α-Glc*p*-(1→4)-α-glycosyl backbone (4, 8). A distinct AB2 pattern of the peaks at δ 4.12 (1H, t, ^3^*J* = 9.1 Hz, >CH-) and a set of two overlapping double doublets at δ 4.07 (2H, dd, ^3^*J* = 9.4 Hz, ^2^*J* = −3.2 Hz, >CH_2_-) were identified (18). Notably, the negative coupling constant was attributed to the two-bond (^2^*J*) coupling between the geminal diastereotopic methylene protons of the glyceric acid residue (19, 20). The NMR analysis suggested that mGP from *M. tuberculosis* has a carbohydrate backbone very similar if not identical to that of other species (5–7, 10, 21).

### The effect of mGP on γ_9_δ_2_ T cells

This mGP was not able to activate γ_9_δ_2_ T cells efficiently when compared with the parent mGLP, indicating that the acyl modifications of the saccharide backbone were necessary for biological activities (Fig. 7*B* illustrates T-cell activity of mGP along with other derivatives). Thus, more detailed biochemical analyses of mGLP were pursued to further identify the nature and modification driving the biological activity.

### Monosaccharide composition of mGLP

The monosaccharide analysis by GC/MS of the G-50–purified mGLP showed three distinct hexoses, 6-*O*-Me-Glc, Glc, and a 3-*O*-Me-Glc in a ratio of 11:8:1 (Fig. S1). Surprisingly, our mGLP preparation did not contain the 2-*N*-acetyl-2,6-dideoxy-β-glucopyranose as in *M. bovis* BCG (10).

### Identification of acyl functions in G-50 purified native mGLP by ^1^H NMR

Native mGLP was analyzed first by ^1^D-^1^H NMR spectroscopy (Fig. 1*B*). The resonances for the acyl substitutions were all evident between δ 0.5 and 2.6 ppm, except in this experiment succinyl resonances could not be unambiguously identified due to overlap issues. The chemical shifts at δ 2.37 (2H, m, H_α_), δ 1.52 (2H, m, H_β_), δ 1.26 (8H, m, H_γ-ζ_), and δ 0.83 ppm (3H, t, ^3^*J* = 6.64 Hz, -Me terminal) provided the evidence for the presence of the octanoyl residue. The chemical shift at δ 2.55 ppm (>CH-) and a set of two overlapping doublets at δ 1.05 (3H, d, ^3^*J* = 7.0 Hz) and δ 1.06 ppm (3H, *d*, ^3^*J* = 7.0 Hz) were attributed to an isobutyryl residue. In addition, three differentially located acetyl groups were at δ 1.99, 2.00, and 2.03 ppm (3 × s, 3 × CH_3_). When the spectrum was integrated with reference to eight protons at δ 1.26 ppm (8H, m, H_γ-η_), the anomeric region accounted for ∼20 protons, suggesting that all the mGLP isoforms contained the octanoyl residue. However, the integral value (∼2.7) exceeds well over two protons at δ 2.37 ppm (2H, m, H_α_), indicating possible overlap from other acyl residues. The relative integral values (the nonoverlapping β-protons (2H) of the octanoyl group at δ 1.52 ppm was the reference integral) revealed an approximate acyl variation ratio (with reference to the octanoyl residue) as octanoyl:isobutyryl:acetyl:succinyl of 1:0.5:0.67:0.35 in the mixture of differentially acylated mGLPs.

### Heteronuclear single quantum coherence (HSQC; ^1^H-^13^C correlation NMR spectroscopy) and total correlation spectroscopy (TOCSY; through-bond ^1^H-^1^H correlation NMR spectroscopy) for confirmation of acyl groups

**Figure 2.**
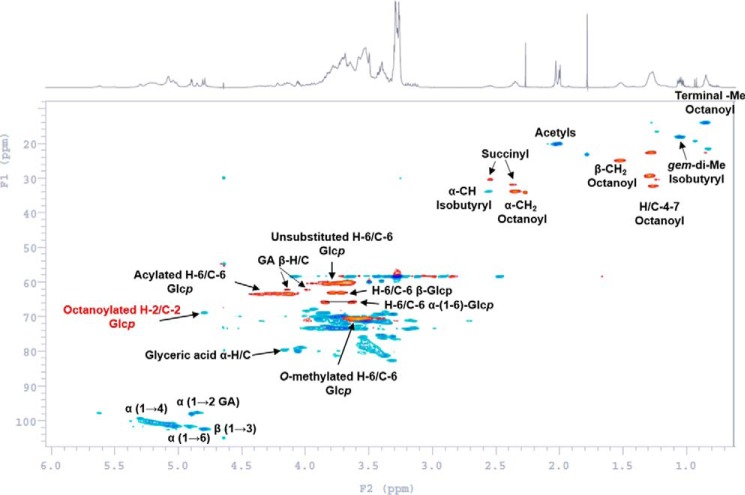
**HSQC NMR (H–C 2D correlation) spectrum of G-50–purified native mGLP.** NMR was performed in D_2_O at room temperature. *Red* contour peaks correspond to methylene (-CH_2_-) groups, and *blue* contour peaks correspond to methyl (-CH_3_) and methine (>CH-) groups. *GA*, glyceric acid.

The HSQC experiment of the G-50–purified mGLP revealed (Fig. 2) the ^13^C resonances at δ 35.0 (C_α_), δ 25.8 (C_β_), δ 29.5 (C_γ_), δ 32.2 (C_δ,ϵ_), δ 22.5 (C_ζ_), and δ 14.0 ppm (Me) for the octanoyl chain. The methylene carbons identified in the HSQC experiment (Fig. 2) at δ 32.0 (C_β_) and δ 30.0 ppm (C_α_) were correlated with the ^1^H spin system in the TOCSY experiment (Fig. S2). As the respective methylene proton's chemical shifts were at δ 2.55 (H_α_) and δ 2.37 ppm (H_β_), we assigned this methylene system to a possible succinyl residue, which was also confirmed by MS. The HSQC experiment also revealed that the methine (>CH-) proton of the isobutyryl residue at δ 2.55 ppm (m, H_α_) overlaps with the methylene proton peak of a succinyl residue. A clear spin system correlation was observed in the TOCSY experiment (Fig. S2 between the peak (>CH- proton at δ 2.55 ppm) and a set of two overlapping doublets at δ 1.05 (3H, d, ^3^*J* = 7.0 Hz) and δ 1.06 ppm (3H, d, ^3^*J* = 7.0 Hz). In the HSQC experiment, the ^13^C for the acetyls clustered around δ 20.0 ppm. As for the glyceric acid residue, the HSQC experiment showed distinct methylene carbon at δ 62.2 ppm (C_β_) with the corresponding protons at δ 4.15 and δ 4.00 ppm, respectively. The carbon centered at δ 79.8 ppm (δ 4.17 ppm (H_α_)) was attributed to C_α_ of the glyceric acid. The different chemical shifts for the diastereotopic protons (H_β_) (giving rise to an ABC pattern; H_α_ and 2 × H_β_) for the glyceric acid has been reported earlier (18).

The HSQC spectra of the mGLP revealed five distinct sets of methylene protons (Fig. 2, in *red*). The protons at δ 3.92–3.60 ppm with corresponding ^13^C chemical shift at δ 60.0–61.0 ppm were attributed to the H-6/C-6 of α-d-Glc*p* unit/s linked through (1→4)-glycosidic bonds (22). The H-6/C-6 of the α-d-6-OMe-Glc*p* units, linked through (1→4)-glycosidic bonds, were assigned to δ 3.71–3.38 ppm with the corresponding ^13^C chemical shift at δ 70.0–71.0 ppm (23). The protons between δ 3.82 and 3.67 ppm with the corresponding ^13^C chemical shift at δ 62.5 ppm was attributed to the H-6/C-6 β-Glc*p* units (24). The ^13^C at δ 66.0 ppm showing two different proton chemical shifts at δ 3.84 and δ 3.63 ppm is possibly due to the diastereotopic relationship and was attributed to the H-6/C-6 of the α-(1→6)-Glc*p*-(1→2)-glyceric acid unit (25, 26). We could assign the ^13^C of the methylene peaks at δ 63.8 ppm with the proton chemical shifts between δ4.42 and 4.22 ppm to the H-6/C-6 of the Glc*p* units that are acyl-substituted. The HSQC experiment revealed a proton at δ 4.8 ppm (>CH-; Fig. 2, in *blue*) with a ^13^C chemical shift at δ 69.5 ppm. We assigned this to the H-2 of a Glc*p* that is likely to be acylated. The only other possibility, *i.e.* H-3 (because H-4 is perhaps glycosyl-linked), would have resulted in a more downfield shift (23, 27) if acylated.

### The nuclear Overhauser effect spectroscopy (NOESY; ^1^H-^1^H correlation NMR spectroscopy) allowing for information on acyl substitution on the carbohydrate backbone

**Figure 3.**
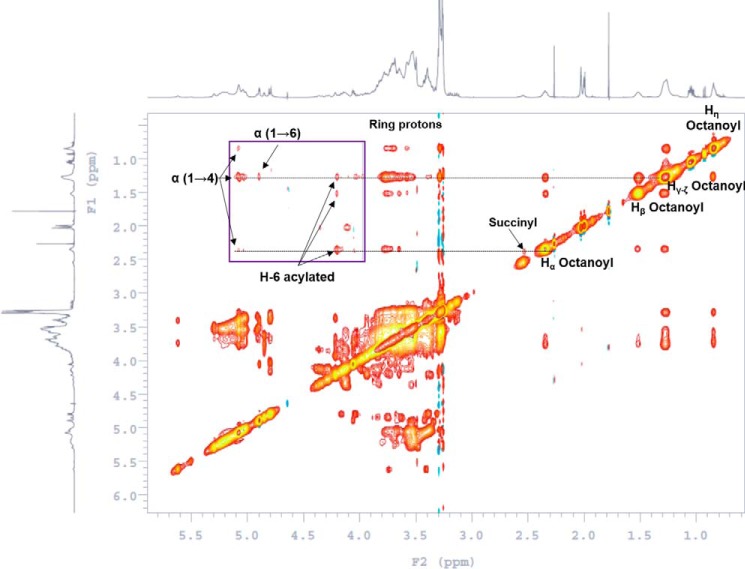
**NOESY of native mGLP (D_2_O; no spin; molecular weight, ∼3800; mixing time, 0.3 s).** Shown is through-space correlation of all protons in the octanoyl residue with the Glc*p* ring protons, succinyl residue (*inset*, magnified δ 4.0–5.2 ppm), and acylated (possibly succinylated) methylene protons. α(1→4) and α(1→6) anomeric protons signify octanoyl as a ring substitution other than C-6 of Glc*p*.

In the NOESY experiment, however, we did not see any through-space coupling of the acetyl or isobutyryl residues with either ring protons (δ 3.0–4.5 ppm) or the anomeric protons (δ 4.6–5.7 ppm) (Fig. 3). This suggested that the acetyl residues and the isobutyryl residue were perhaps attached to the primary carbon (C-6 position) of Glc*p* units. However, the octanoyl residue showed through-space correlation not only with Glc*p* ring protons at δ 3.53–3.72 ppm but also with the anomeric protons at δ 5.02–5.12 (α-(1→4)-Glc*p*) and δ 4.90 ppm (α-(1→6)-Glc*p*)), indicating that the octanoyl chain is located on the C-2 position of α-Glc*p* at the reducing end. Furthermore, the α-protons of the octanoyl residue showed specific NOE correlations with protons at δ 2.55 ppm (possibly H_α_ of a succinyl residue (correlation with isobutyryl was ruled out because the pattern would have been different)) and acetylated methylene (-CH_2_-) protons at δ 4.20 ppm in addition to anomeric protons at δ 5.02–5.12 ppm (α-(1→4)-Glc*p*). This suggested that the octanoyl residue is possibly attached to the C-2 of a glucosyl residue with a succinyl substitution close by as in 6-substituted β(1→3)-Glc*p* (5, 28, 29) and not on the glyceric acid as has been reported in the past (4). Characterization of all the acyl groups present in mGLP are listed in Table 1.

**Table 1:**
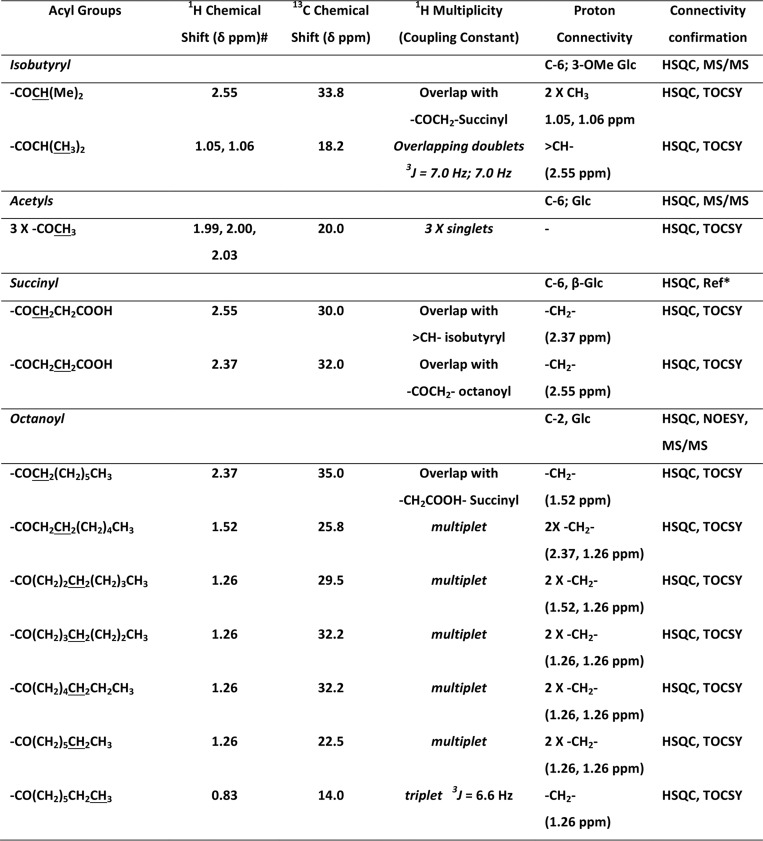
**Characterization of acyl functions of mGLP** HSQC, ^1^H-^13^C correlation NMR spectroscopy; TOCSY, total ^1^H-^1^H correlation NMR spectroscopy; NOESY, nuclear Overhauser effect NMR spectroscopy: Through space ^1^H-^1^H correlation; MS/MS, tandem mass spectroscopy.

* Refs. 5 and 28.

### LC/MS analysis of G-50 purified native mGLP

At first, a direct infusion of G-50–purified native mGLP in the ESI-MS negative mode showed a singly charged cluster of ions at mass-to-charge ratio (*m*/*z*) 1033, identified as a contaminant and [M − 2H]^2−^ at *m*/*z* 1918.2, and triply charged dominant species at *m*/*z* 1278.5 [M − 3H]^3−^, which agreed with the calculated molecular weight of mGLP based on published structural studies (4) of 3838.61. This molecular weight of mGLP corresponded to 12 *O*-methylated Glc, eight Glc, one glyceric acid, and acyl groups comprising three acetyls, one octyl, and one isobutyryl. The spectra of the mGLP also revealed that the preparation was contaminated with trace amounts of lysophosphatidyl dimannoside at *m*/*z* 895.39 and 987.44 (30).

**Figure 4.**
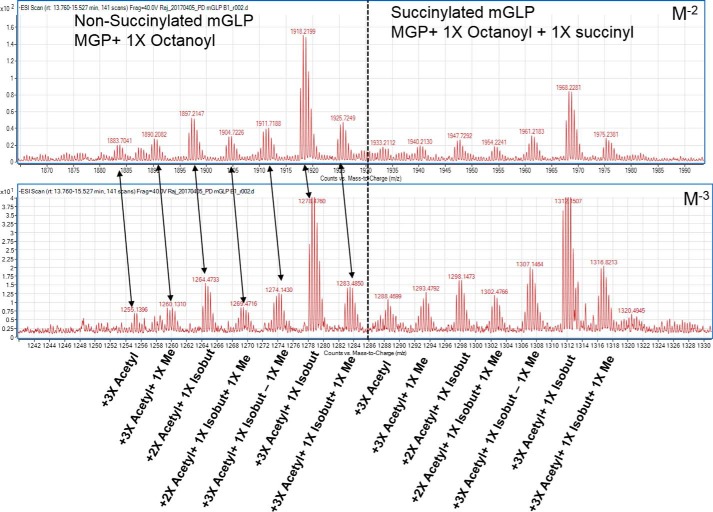
**LC/MS (negative ionization) of native mGLP.** A reverse-phase C_18_ column with NH_4_OAc:CH_3_CN gradient was used to resolve the isoforms. Each ion cluster corresponds to one isoform of mGLP (altogether 14 isoforms; seven nonsuccinylated and seven succinylated). The *upper panel* represents [M − 2H]^2−^ ions, and the *lower panel* represents corresponding [M − 3H]^3−^ ions. *isobut*, isobutyryl.

We reasoned that the mGLP could be further resolved into uniform acetylated forms, and these separated forms could then be analyzed with MS/MS for the precise location of the acyl groups. The G-50–purified mGLP was subjected to LC/MS with an ammonium acetate and acetonitrile linear gradient and readily yielded the doubly and triply deprotonated anions [M − 2H]^2−^ and [M − 3H]^3−^. Overall, 14 differentially functionalized mGLP isoforms were identified (Fig. 4 and Table 2). Among these 14 forms, seven were nonsuccinylated (i–vii), and the same seven were found to be modified with a single succinyl residue (ia–viia; molecular mass, +100 Da). The major acyl forms (iii and iiia) of mGLP, as evident from relatively higher abundance of ions (Fig. 4), were found to have three acetyls, one isobutyryl, one octanoyl with and without a succinyl residue. The other acyl forms were missing either an acetyl or isobutyryl group and/or an extra methyl group as detailed in Table 2.

**Table 2:**
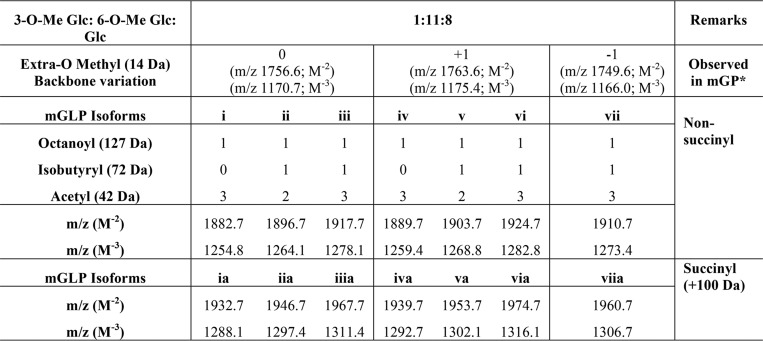
**Acyl modifications and heterogeneity on the carbohydrate backbone-analysis by LC/MS**

* See Fig. S4.

### Glycomic and acylation profile of diacetylated mGLP (molecular weight, 3795) by QTOF ESI-MS/MS

**Figure 5.**
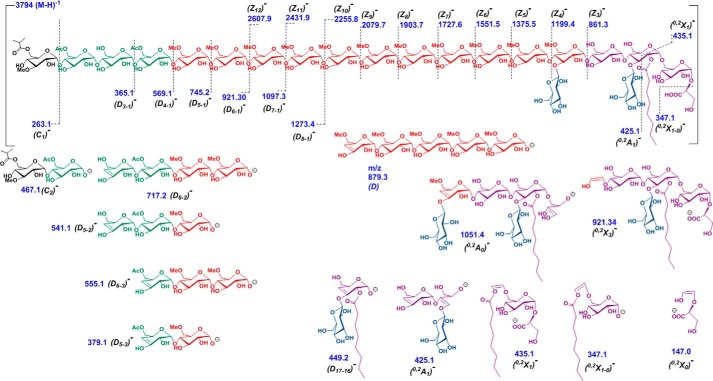
**Tandem mass spectroscopic analysis of mGLP.** ESI-collision-induced dissociation in negative ion mode shows 80- and 100-eV fragment ions [M − H]^−^ of LC-purified native mGLP isoform (molecular weight, 3795; *m*/*z* 1264.1 [M − 3H]^3−^). *Z_i_* and *X_i_* ions correspond to the number of glycosidic linkages from the reducing end; *C_i_*, *D_i_*, and *A_i_* ions correspond to the number of glycosidic linkages from the nonreducing end. The fragment ions suggested the locations of isobutyryl, acetyl, octanoyl, and glyceric acid residues and β-d-Glc*p*-(1→3) branches on the mGLP skeleton.

Identification of the site of acyl modification in mGLP was sought using tandem MS of native mGLP. The triply charged ions at *m*/*z* 1264.1, with a mass corresponding to a composition of one 3-*O*Me-Glc, 11 6-*O*Me-Glc, eight Glc, two acetyls, one isobutyryl, one octanoyl, and one glyceric acid residue was subjected to MS/MS fragmentation in negative ion (ESI) mode using collision-induced dissociation (the ions and structures are presented in Fig. 5, and the mass spectra are presented in Fig. S4, *A–E*). Collision energy was optimized to be 80 and 100 eV. A charge-reduced mass fragmentation pattern was observed whereby singly charged [M − H]^−^ product ions were obtained (31).

The nonreducing end *m*/*z* 263.1 (*C*_1_)^−^ fragment and the corresponding *m*/*z* 245.1 (*B*_1_)^−^ fragment account for a 3-*O*-Me-glucosyl residue substituted with one isobutyryl residue. The *m*/*z* 467.1 (*C*_2_)^−^ fragment (see Fig. 5) has the correct mass of a diglucosyl with one methyl, one isobutyryl, and one acetyl residue, signifying that one acetyl function is located on the second Glc unit of the nonreducing end. Additional information on the sequence at the nonreducing end of the molecule came from the ions produced by double glycosidic cleavages. Such cleavages are found in nonderivatized oligosaccharides when subjected to negative ESI-tandem MS and are labeled with *D* in Fig. 5 and Fig. S4, *A–E*. The subscripts indicate the two cleavage glycosidic bonds counting from the nonreducing end (32). The double cleavage ions where the 3-*O*-Me-glucosyl residue (residue 1) is lost proved particularly informative. The *m*/*z* 1273.4 (*D*_8-1_)^−^, 1097.3 (*D*_7-1_)^−^, 921.30 (*D*_6-1_)^−^, 745.2 (*D*_5-1_)^−^, and 569.1 (*D*_4-1_)^−^ fragments all contain three nonmethylated glucosyl units with four, three, two, and zero 6-*O-*Me-glucosyl residues. The *m*/*z* 569.1 (*D*_4-1_)^−^ fragment corresponded to three nonmethylated glucosyl residues with two acetyl groups. Given the fact that the second Glc has an acetyl unit, the additional acetyl must be either on the third or fourth Glc unit. The ions at *m*/*z* 379.1 (*D*_5-3_)^−^ and 555.1 (*D*_5-2_)^−^ show that the second acetyl is on the fourth glucosyl residue. The ions at *m*/*z* 365.1 (*D*_3-1_)^−^ and 717.2 (*D*_6-2_)^−^ are consistent with this assignment. These fragments (along with the NMR analysis showing that the acetyl groups are on the 6-position) account for a sequence of octaglucoside of the nonreducing end of mGLP of α-d-Glc*p*(3Me)(6isobutyryl)-(1→4)-α-d-Glc*p*(6Ac)-(1→4)-α-d-Glc*p-*(1→4)-α-d-Glc*p*(6Ac)-(1→4)-α-d-Glc*p*(6Me)-(1→4)-α-d-Glc*p*(6Me)-(1→4)-α-d-Glc*p*(6Me)-(1→4)-α-d-Glc*p*(6Me).

The reducing end *Z* fragments (*Z* and *X* ions are numbered from the reducing end of the molecule) from *m*/*z* 2607.9 (*Z*_12_)^−^ down to *m*/*z* 1199.4 in 176 Da shows the presence of eight additional unbranched 6-*O*-methylglucosyl units toward the reducing end from the nonreducing octaglucoside just described. If the next 6-*O*-methylglucoside was also unbranched, an ion at *m*/*z* 1023.3 should be present; however, this ion is missing. Instead the *Z*_3_^−^ ion is present 861.3, showing that the reducing end 6-*O*-methylglucosyl unit (the fourth main-chain glucosyl from the reducing end) is branched with a single glucosyl unit. These leave a reducing end with four non-*O*-methylated glucosyl residues, one octanoyl, and one glyceric acid residue. The ions at of *m*/*z* 449.1 (*D*_17-16_)^−^ and 347.1 (^0,2^*X*_1_*-*glyceric acid) contain the octanoyl group but not the glyceric acid group and thus show that the octanoyl group cannot be attached to the glyceric acid. The HSQC and NOESY NMR experiments showed that the octanoyl group is attached to the 2-position of a glucosyl unit (Fig. 2), further substantiated by an ion at *m*/*z* 435.1 (^0,2^*X*_1_)^−^. The double cleave ion at *m*/*z* 1051 (^0,2^*A*_0;2;4_)^−^, which contains the octanoyl group along with the branched 6-*O*-methylglucosyl residue, both β-glucosyl residues, a linear glucosyl residue, and the O-3, O-4, O-5, and O-6 oxygens of the glucosyl residue at the reducing end (but not O-2), rules out the possibility of the octanoyl group being attached to the first glucosyl residue. This conclusion is confirmed by the ion at *m*/*z* 147 (^0,2^*X*_0_)^−^, which contains O-2 of the first glucosyl residue but no octanoyl group. Therefore the entire mGLP has the following sequence: α-d-Glc*p*(3Me)(6isobutyryl)-(1→4)-α-d-Glc*p*(6Ac)-(1→4)-α-d-Glc*p-*(1→4)-α-d-Glc*p*(6Ac)-(1→4)-α-d-Glc*p*(6Me)-(1→4)-α-d-Glc*p*(6Me)-(1→4)-α-d-Glc*p*(6Me)-(1→4)-α-d-Glc*p*(6Me)-(1→4)-α-d-Glc*p*(6Me)-(1→4)-α-d-Glc*p*(6Me)-(1→4)-α-d-Glc*p*(6Me)-(1→4)-α-d-Glc*p*(6Me)-(1→4)-α-d-Glc*p*(6Me)-(1→4)-α-d-Glc*p*(6Me)-(1→4)-α-d-Glc*p*(6Me)-(1→4)-α-d-Glc*p*(6Me)-(1→4)-α-d-Glc*p*(6Me)[β-d-Glc*p-*(1→3)]-(1→4)-α-d-Glc*p*(6Me)-(1→4)-α-d-Glc*p*[β-d-Glc*p-*(1→3)](2octanoyl)-(1→6)-α-d-Glc*p-*(1→2)-glyceric acid).

### Structural and biological analysis of α-amylase–treated mGLP

**Figure 6.**
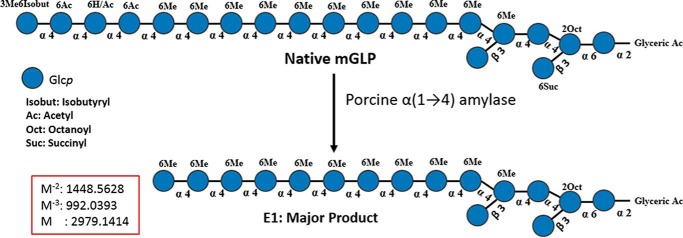
**Representation of native mGLP and its enzymatic porcine α(1→4)-amylase) digestion product.** The MS of the major product (Fig. S5) isolated corresponded to the above structure drawn of the reducing end of mGLP (with glyceric acid and octanoyl groups intact) arising after the enzymatic cleavage of three Glc*p* units plus one Glc*p*(3Me) unit from the nonreducing end carrying isobutyryl and acetyl residues.

**Figure 7.**
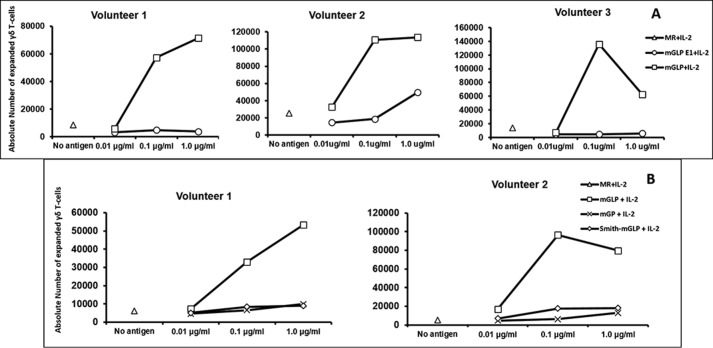
**γ_9_δ_2_ T-cell expansion profile of mGLP derivatives with different human PBMC volunteers.** *A*, concentration (0.01, 0.1, and 1.0 μg/ml)-wise γ_9_δ_2_ T-cell expansion profile of mGLP derivatives (absolute numbers of expanded T cells with three volunteers). ▵, medium-rested (*MR*) + interleukin 2 (IL-2) is the baseline control in the absence of any antigen. □, native mGLP + IL-2 showed the best expansion ability at 0.1 μg/ml; saturation of biological response may be responsible for a dip in expanded T-cell numbers at 1.0 μg/ml. The enzyme-digested product (○) mGLP E1 + IL-2, which lost four nonreducing-end hexoses, two to three acetyls, and one isobutyryl group, showed inability for T-cell expansion at 0.01 or 0.1 μg/ml but a very weak expansion at 1.0 μg/ml. *B*, concentration (0.01, 0.1, and 1.0 μg/ml)-wise γ_9_δ_2_ T-cell expansion profile of mGLP derivatives (absolute numbers of expanded T-cells with two volunteers). ▵, medium-rested (*MR*) + IL-2 is the baseline control in the absence of any antigen. □, native mGLP + IL-2 showed the best expansion ability at 0.1 μg/ml. ×, mGP + IL-2 showed inability for T-cell expansion at 0.01 or 0.1 μg/ml but a very weak expansion at 1.0 μg/ml. ♢, the Smith degraded product from mGLP + IL-2 showed inability for T-cell expansion at 0.01 or 0.1 μg/ml but a very weak expansion at 1.0 μg/ml.

Exhaustive digestion of native mGLP with porcine α-amylase yielded one enzyme major limit product, E1 (Fig. 6), which was characterized by subsequent LC/MS, revealing *m*/*z* 1489.06 [M − 2H]^2−^ (Fig. S5). This mass was attributed to the sequence α-d-Glc*p*(6Me)-(1→4)-α-d-Glc*p*(6Me)-(1→4)-α-d-Glc*p*(6Me)-(1→4)-α-d-Glc*p*(6Me)-(1→4)-α-d-Glc*p*(6Me)-(1→4)-α-d-Glc*p*(6Me)-(1→4)-α-d-Glc*p*(6Me)-(1→4)-α-d-Glc*p*(6Me)-(1→4)-α-d-Glc*p*(6Me)-(1→4)-α-d-Glc*p*(6Me)-(1→4)-α-d-Glc*p*(6Me)-(1→4)-α-d-Glc*p*(6Me)[β-d-Glc*p-*(1→3)]-(1→4)-α-d-Glc*p*(6Me)-(1→4)-α-d-Glc*p*[β-d-Glc*p-*(1→3)](2octanoyl)-(1→6)-α-d-Glc*p-*(1→2)-glyceric acid). This indicated that 6-*O*-Me-Glc units are responsible for enzyme resistance. Notably, the succinyl residue, along with all the acyls but octanoyl, were lost during the digestion. The product E1 was purified from the digest mixture and tested for its ability to activate γ_9_δ_2_ T cells. Compared with native mGLP, a ∼90% loss in γ_9_δ_2_ T-cell expansion ability was associated with E1 (Fig. 7*A*).

## Discussion

BCG vaccine is primarily used against tuberculosis, particularly in the endemic countries. BCG vaccines, comprising attenuated *M. bovis* strains, are the only vaccines known to induce protective immunity even when given to infants at birth. Despite these potent stimulatory capacities of *M. tuberculosis* and BCG, a third of the world is latently infected with *M. tuberculosis*, and ∼1.5 million people die annually from TB disease complications. Recent TB vaccine development efforts have focused almost exclusively on the induction of αβ T cells specific for immunodominant peptide epitopes, perhaps only enhancing the evolutionary advantage for the pathogen. It certainly remains possible that induction of the right combination of αβ T-cell antigen specificity and effector phenotypes will result in more successful TB vaccines. However, the current goals also involve development of novel vaccine strategies and targets in tandem with further characterization of nonconventional T cells (33), including protective immune subsets such as γ_9_δ_2_ T cells. The γ_9_δ_2_ (also termed Vγ_9_Vδ_2_) T cells provide a natural bridge between innate and adaptive immunity, rapidly and potently respond to pathogen infection in mucosal tissues, and are prominently induced by both TB infection and BCG vaccination. Therefore, these cells may serve as potent targets for TB immunotherapy. Recent work has demonstrated important TB-protective effects of γ_9_δ_2_ T cells against intracellular replication of mycobacteria in both nonhuman primates and human systems (34, 35). It has been shown that nonhuman primates develop protective memory γ_9_δ_2_ T cells after BCG vaccination, and this has greatly contributed to our knowledge of how these TB-protective γ_9_δ_2_ T cells function *in vivo* and protect against primary TB and other infections (36–42). Our group has shown that BCG vaccination in humans induces γ_9_δ_2_ memory T cells (35, 43, 44) and that these γ_9_δ_2_ T cells develop pathogen specificity (43, 44), can potently inhibit intracellular mycobacterial growth (44), and utilize a novel protective mechanism to inhibit intracellular *M. tuberculosis* (45). In addition, we have now described that mGLP from *M. tuberculosis*, and not simple phosphoantigens previously shown to activate all γ_9_δ_2_ T cells, can expand a protective subset of γ_9_δ_2_ T cells. The mGLP-induced T-cell subset expresses a restricted subset of receptor (TCR) sequences. Unlike αβ T cells, γδ T cells do not require antigen processing prior to recognition of certain bacterial, lipid, and tumor antigens. Recognition of these T cells by mGLP is a novel finding, and the mechanism is unclear. mGLP is not a major component of *M. tuberculosis*; in fact, we obtained ∼200 mg of purified mGLP from 2500 g of biomass (0.01%). Despite the presence of a number of glycosyl residues, the molecule is somewhat hydrophobic and acylated, making it behave like a lipid rather than a glycan. The octanoyl group in mGLP has been suggested to have a specific role in stabilizing the polysaccharide in helical conformation, providing it with further discriminatory capability when binding fatty-CoAs, and anchoring mGLP intermediates to the cytoplasmic membrane during the elongation steps (6, 46). Due to stereochemical constraints arising from the α-(1→4) linkages in the main chain, mGLP likely adopts a helical conformation in solution with the methyl groups facing the inner cylindrical hydrophobic cavity (5).

In an effort to establish a structure-to-function relativity, we took a stepdown approach in dissecting mGLP and testing for biological potency. Thus far, we were able to show that Smith degradation of mGLP, leading to the formation of a polyol, was unable to expand γ_9_δ_2_ T cells (Fig. 7*B*). Following this, the product obtained after mild alkali treatment (mGP; NMR showing the absence of any acyl functions) could only weakly expand T cells, indicating that in some fashion the acyl groups contributed to the biological interplay of mGLP with γ_9_δ_2_ T cells. Next, we treated mGLP with porcine α-amylase. The major acidic product isolated after size-exclusion chromatography was analyzed and yielded one major doubly charged product with *m*/*z* 1489.06 [M − 2H]^2−^, confirming it to be an oligomer comprising 11 methyl-Glc, five Glc, one glyceric acid, and one octanoyl residue (Fig. 6). This enzyme-resistant product was also unable to induce γ_9_δ_2_ T cells. These results prompted us to conclude that all or some of the small acyl residues located at the nonreducing end of the molecule prior to the assembly of the 6-*O*-methyl-Glc–containing glycan segment are responsible for the TB-specific γ_9_δ_2_ T cell–inducing biologic activity. Because of the involvement of the acyl groups in the specific biological activity, we set out to identify the acyl group at each position of esterification. It has been shown clearly that acyl functions were located at the terminal 3-*O*-methyl-Glc end, and the glyceric acid and octanoyl group are at the reducing end of the molecule (6).

For current analyses, we relied on 2D NMR and ESI-tandem MS. The questions we sought to answer were: (*a*) whether the five small acyl groups in mGLP (three acetyls, one isobutyryl, and one succinyl) influence T-cell recognition, (*b*) how are these distributed throughout the molecule, and (*c*) what is the degree of heterogeneity. Due to the complexity and heterogeneity in the molecule, we used one dominant LC-purified homogeneous isoform of molecular weight 3795 (*m*/*z* 1264.1 [M − 3H]^3−^; composition, diacylated mGLP), for a detailed MS/MS experiment by sensitive ultrahigh-resolution QTOF MS/MS. A detailed fragmentation analysis indicated the two acetates to be on the 6-position of the nonreducing-end Glc*p*(s), and NMR and MS analyses suggest that, in the *M. tuberculosis* mGLP, the octanoyl group is at the C-2 position of the second Glc*p* from the reducing end. To summarize, from the structure/function relationship studies, it is now possible to build upon this knowledge such that synthetic intermediates can be generated in large amounts for *in vivo* application.

## Experimental procedures

### Materials

Sephadex G-50 (fine) was obtained from Sigma-Aldrich. All reagents for biochemical separations were obtained from Acros Organics (silica gel 60) and Sigma-Aldrich (chloroform and methanol).

### Isolation of mGLP

Large-scale mid-log mycobacterial cultures (*M. tuberculosis* H37Rv) were harvested and washed twice with sterile H_2_O. The pellet was lyophilized and extracted with chloroform:methanol:H_2_O (10:10:3, v/v/v) twice at room temperature. The organic phase was dried under N_2_ and stored at −20 °C. Total lipid extracts were fractionated over silica gel 60 (EM Science, Fort Washington, PA) using increasing amounts of methanol in chloroform. The 100% methanol eluent was found to be enriched with mGLP (16) and taken for further resolution.

### Purification of mGLP

100% CH_3_OH fraction (12 mg of carbohydrate) was dissolved in water (0.5 ml), applied onto the size-exclusion column (Sephadex G-50; 114 × 0.75 cm), and eluted with water. The flow rate was maintained at 0.55 ml/min. Fractions (120; each 2.5 ml/fraction/5.0 min) were collected. A quick α-naphthol charring thin-layer chromatography (TLC) assay was performed to identify the carbohydrate-enriched (25th to 43th; 62.5–107 ml) fractions. Every three consecutive fractions were then pooled, and the monosaccharide composition was determined after derivatization using GC/MS. The analysis revealed that the 62.5–70-ml fraction had the enriched mGLP (without detectable impurities by ^1^H NMR). The overall purification and characterization strategy is presented as a flow sheet in Fig. S6.

### Deacylation of mGLP

G-50–purified mGLP (1.0 mg) was dissolved in 0.2 n NaOH (1 ml) and allowed to remain at 55 °C for 2 h. The solution was then neutralized with acetic acid, dried, and applied to a Bio-Gel P-2 column (0.5 × 50 cm) in water for desalting. The deacylated product (mGP) was checked by ^1^H NMR to ensure completion of deacylation.

### Porcine α-amylase treatment

mGP (1.0 mg) and mGLP (3.0 mg) in phosphate-buffered saline (PBS; pH 7.2) were treated simultaneously with porcine pancreatic α-amylase (Sigma; 3 and 6 units) for 24 and 72 h, respectively, at 37 °C. TLC on silica gel 60 plates (Merck) with a solvent system composed of chloroform:methanol:water (56:38:10, v/v/v) was used to monitor enzyme activity (formation of new products and utilization of starting material), visualized by spraying with α-naphthol–sulfuric acid solution followed by charring at 120 °C. Whereas mGP was digested fully, only partial change was observed with mGLP. Enzyme was deactivated, and the digestion mixture was desalted on a Bio-Gel P-2 column (0.5 × 30 cm) followed by a G-50 column. The α-naphthol–positive fractions were used for downstream analyses.

### Monosaccharide composition

Aliquots of G-50 column eluents were hydrolyzed with 2 m TFA, converted to alditol acetates, and analyzed using GC/MS performed as described previously (47).

### 1D and 2D NMR analyses

All PRESAT ^1^H NMR was recorded in D_2_O on a 400-MHz Innova (Varian), and 2D NMR (TOCSY, C2HSQC, and NOESY) was recorded on a 500-MHz Innova (Varian) instrument at 25 °C. All chemical shifts are based on the reference to the HOD peak at δ 4.64 ppm. The default Varian parameters were used for recording spectra.

### Liquid chromatography time-of-flight MS

#### 

##### Accurate mass LC/MS analyses

LC/MS was performed on an Agilent 1260 Infinity series HPLC in line with a 6224 time of flight (TOF) MS equipped with a multimode ESI/atmospheric pressure chemical ionization source operated in negative ESI mode. Gradient separation of a 0.4 mg/ml solution of mGLP in water was performed over an HPLC column (Waters X-Bridge C_18_, 2.1 × 150 mm, 3.5-μm particle size) held at 40 °C with a consistent 0.32 ml/min flow rate. Injections were 2 μl, and all solvents were LC/MS grade (Fisher Optima). Starting conditions, 90% solvent A (H_2_O with 10 mm ammonium acetate), 10% solvent B (acetonitrile with 10 mm ammonium acetate), were held for 5 min, then increased to 70% B over 10 min in a linear gradient followed by an increase to 100% B over 1 min, and then held for 4 min as a wash step. MS instrument parameter settings were as follows: gas temperature, 310 °C; vaporizer temperature, 200 °C; gas flow, 10 ml/min; nebulizer pressure, 45 p.s.i. gauge; and charging voltage, 2000 V. MS source parameter settings were set as follows: capillary voltage, 2500 V; fragmentor, 40; and skimmer1, 60.

##### Liquid chromatography quadrupole time-of-flight MS

Structural elucidation of the mGLP was carried out by ultraperformance LC (UPLC) on a Waters Acquity UPLC H-Class system coupled to a Bruker MaXis Plus QTOF MS instrument. Separation was performed in gradient mode with a Waters Acquity UPLC BEH C_18_ 1.7-μm column (2.1 × 50 mm) at 40 °C. Mobile phase components were 10 mm ammonium acetate in water (A) and acetonitrile (B). The flow rate was 0.4 ml/min. The proportion of acetonitrile was increased from 10 to 70% in 3 min and then to 100% in 3.4 min and held at 100% for 1.4 min. The post-time was 2 min, and the injection volume was 3 μl. Internal instrument mass-scale calibration was performed in enhanced quadratic mode during chromatographic dead time by infusing Agilent ESI-L low concentration tuning mix.

Data acquisition in negative electrospray ion mode with an *m*/*z* range of 110–4000 at 1 Hz was performed in full-MS scan mode for the first 2 min, during which the internal calibrant was introduced into the LC flow. This was followed by a 1-min multiple reaction monitoring scan mode alternating collision-induced dissociation energies of 6 and 40 eV on *m*/*z* 1264.1 [M − 3H]^3−^ parent ion with *m*/*z* width of 0 and 6, respectively. The final 3.8 min were in full-MS scan mode. Source settings for all time segments were as follows: capillary voltage, 2400 V; end-plate offset, 500 V; nebulizer gas pressure, 3 bar; drying gas flow, 10 liters/min; and drying temperature, 300 °C. Instrument controls were performed via the Bruker HyStar v4.1 software package. Data were processed using Bruker Compass 2.0 Data Analysis 4.4 software.

### γ_9_δ_2_ T cell–stimulatory activity

The assay was performed as described previously (16). Briefly, to expand γ_9_δ_2_ T cells, isolated PBMCs (1 × 10^6^) were cultured with novel antigen fractions or controls (medium-rested and 20 μg/ml *M. tuberculosis* whole-cell lysate). On day 7, the PBMCs were harvested, counted, stained with anti-γδTCR (clone 11F2), anti-αβTCR (clone B3), and anti-CD3 peridinin chlorophyll protein (PerCP) (clone SK7). Absolute numbers of γ_9_δ_2_ T cells were computed by multiplying the flow cytometric percentages by the numbers of viable cells present after expansion. Expansion indices were calculated as the -fold expansion of the absolute number of γ_9_δ_2_ T cells after stimulation with treated lysates compared with the absolute number of γ_9_δ_2_ T cells after rest in medium.

## Author contributions

P. D., M. X., and C. R. formal analysis; P. D., M. M., C. M. B., K. M. D., D. H., and D. C. validation; P. D., M. M., M. X., D. C. H., K. D., T. S., and K. M. D. methodology; P. D., M. M., C. M. B., K. D., K. M. D., and D. H. writing-review and editing; M. M., C. R., and D. C. supervision; C. M. B., D. C. H., K. D., C. R., and T. S. resources; K. D. software; C. R. and D. C. visualization; K. M. D., D. H., and D. C. funding acquisition; K. M. D., D. H., and D. C. project administration; D. H. investigation; D. C. conceptualization; D. C. writing-original draft; M. X. γδ T-cell analyses.

## Supplementary Material

Supporting Information

## Footnotes

2 The abbreviations used are:

mGLP: 6-*O*-methylglucose–containing lipopolysaccharide
mGP: deacylated mGLP
ESI: electrospray ionization
BCG: bacillus Calmette-Guérin
QTOF: quadrupole TOF
HSQC: heteronuclear single quantum coherence
TOCSY: total correlation spectroscopy
MS/MS: tandem mass spectroscopy
TB: tuberculosis
TCR: T-cell receptor
PRESAT: presaturation
C2HSQC: doubly compensated HSQC
UPLC: ultraperformance LC
PBMC: peripheral blood mononuclear cell.
