# Supplementary material for: Structural determinants in a glucose-containing lipopolysaccharide from Mycobacterium tuberculosis critical for inducing a subset of protective T cells
Source: J Biol Chem. 2018 May 1;293(25):9706–17. doi: 10.1074/jbc.RA118.002582 (PMC6016469; doi:10.1074/jbc.RA118.002582)
Supplement: Supporting Information [file supp_293_25_9706__index.html]

Structural determinants in a glucose-containing lipopolysaccharide from Mycobacterium tuberculosis critical for inducing a subset of protective T cells — Structural and functional diversity in mGLP — Structural determinants in a glucose-containing lipopolysaccharide from Mycobacterium tuberculosis critical for inducing a subset of protective T cells — Structural and functional diversity in mGLP — Supporting Information 

# Structural determinants in a glucose-containing lipopolysaccharide from *Mycobacterium tuberculosis* critical for inducing a subset of protective T cells

## Supporting Information

- Structural determinants in a glucose-containing lipopolysaccharide from Mycobacterium tuberculosis critical for inducing a subset of protective T cells - Supporting NMR and MAss spectral data for online
