# Supplementary material for: Structural determinants in a glucose-containing lipopolysaccharide from Mycobacterium tuberculosis critical for inducing a subset of protective T cells
Source: J Biol Chem. 2018 May 1;293(25):9706–17. doi: 10.1074/jbc.RA118.002582 (PMC6016469; doi:10.1074/jbc.RA118.002582)
Supplement: Supporting Information [file supp_RA118.002582_136232_2_supp_130179_p80hz1.docx]

**Structural determinants in a glucose-containing lipopolysaccharide from *Mycobacterium tuberculosis* critical for inducing a subset of protective T cells**

Prithwiraj De^1^, Michael McNeil^1^, Mei Xia^3^, Claudia Boot^2^, Danny C. Hesser^1^, Karolien Denef^2^, Christopher Rithner^2^, Tyler Sours^2^, Karen M. Dobos^1^, Daniel Hoft^3^, and Delphi Chatterjee^1^

^1^Mycobacteria Research Laboratories, Department of Microbiology, Immunology and Pathology, Colorado State University, Fort Collins, CO 80523, USA.

^2^Central Instrument Facility, Department of Chemistry, Fort Collins, CO 80523, USA.

^3^Departments of Internal Medicine, Saint Louis University, St. Louis, Missouri 63104

Running Title: *Structural Diversity in mGLP*

**Contents**

| **Fig S-1** | Chromatograms of monosaccharide analysis of G-50 fractions of mGLP by alditol acetate- GC/MS method. | **Page S-2** |
| --- | --- | --- |
| **Fig S-2** | *The Total Correlation Spectroscopy (TOCSY) of native mGLP: Through bond correlation of acyl functionalities present in mGLP.* | **Page S-3** |
| **Fig S-3** | *LC/MS (negative ionization; M-2) of deacyl mGLP (mGP): each ion cluster corresponds to one isoform of mGLP. Additional methyl group (mGP + 1X Me) may indicate biosynthetic chain termination polysaccharide and mGP- 1X Me may represent under-methylated polysaccharide variation of mGLP backbone.* | **Page S-3** |
| **Fig S-4A** | *MS/MS fragments:m/z 1350-m/z 2620* | **Page S-4** |
| **Fig S-4B** | *MS/MS fragments:m/z 620-m/z 1400* | **Page S-4** |
| **Fig S-4C** | *MS/MS fragments:m/z 320-m/z 720; m/z 425.1* | **Page S-5** |
| **Fig S-4D** | *MS/MS fragments: m/z 921.30, 921.34; m/z 435.14, m/z 347.13* | **Page S-5** |
| **Fig S-4E** | *MS/MS fragments: m/z 147.0 & 1051.37* | **Page S-6** |
| **Fig S-5** | *The MS of the major product and its plausible structure obtained from porcine amylase treatment of naive mGLP.* | **Page S-7** |
| **Fig S-6** | *Overall purification and characterization strategy for Mtb mGLP.* | **Page S-8** |

Abbreviations: mGLP, 6-O-methylglucolipopolysaccharide; mGP, 6-O-methylglucopolysaccharide; lc-ms, liquid chromatography-mass spectrometry; ESI, Electro spray ionization; NMR, Nuclear Magnetic Resonance;


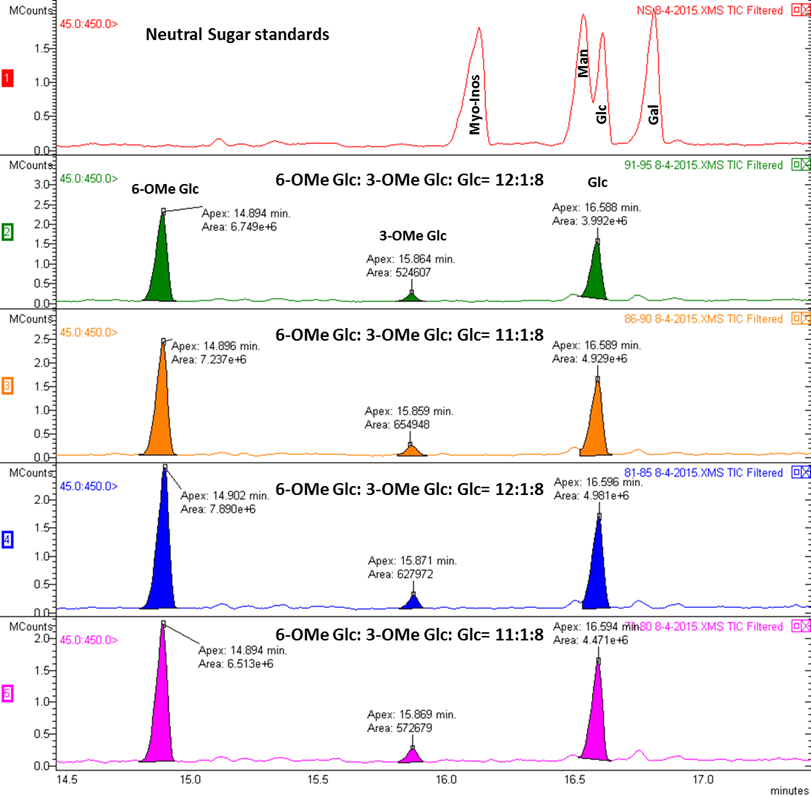


**Fig S-1:** **Chromatograms of monosaccharide analysis of G-50 fractions of mGLP by alditol acetate- GC/MS method.** Panel 1: neutral sugar standards: myo-Inositol (not a sugar) Mannose, Glucose and galactose. Panel 2 through Panel 5: Fractions (77-95; pooled 3 fractions each) out of G-50 column chromatography: only 3 different hexoses were found, 6-OMe Glc, 3-OMe Glc and Glc. They varied by the area ratio 11:1:8 and 12: 1: 8. Area selection was processed manually for integration purpose.


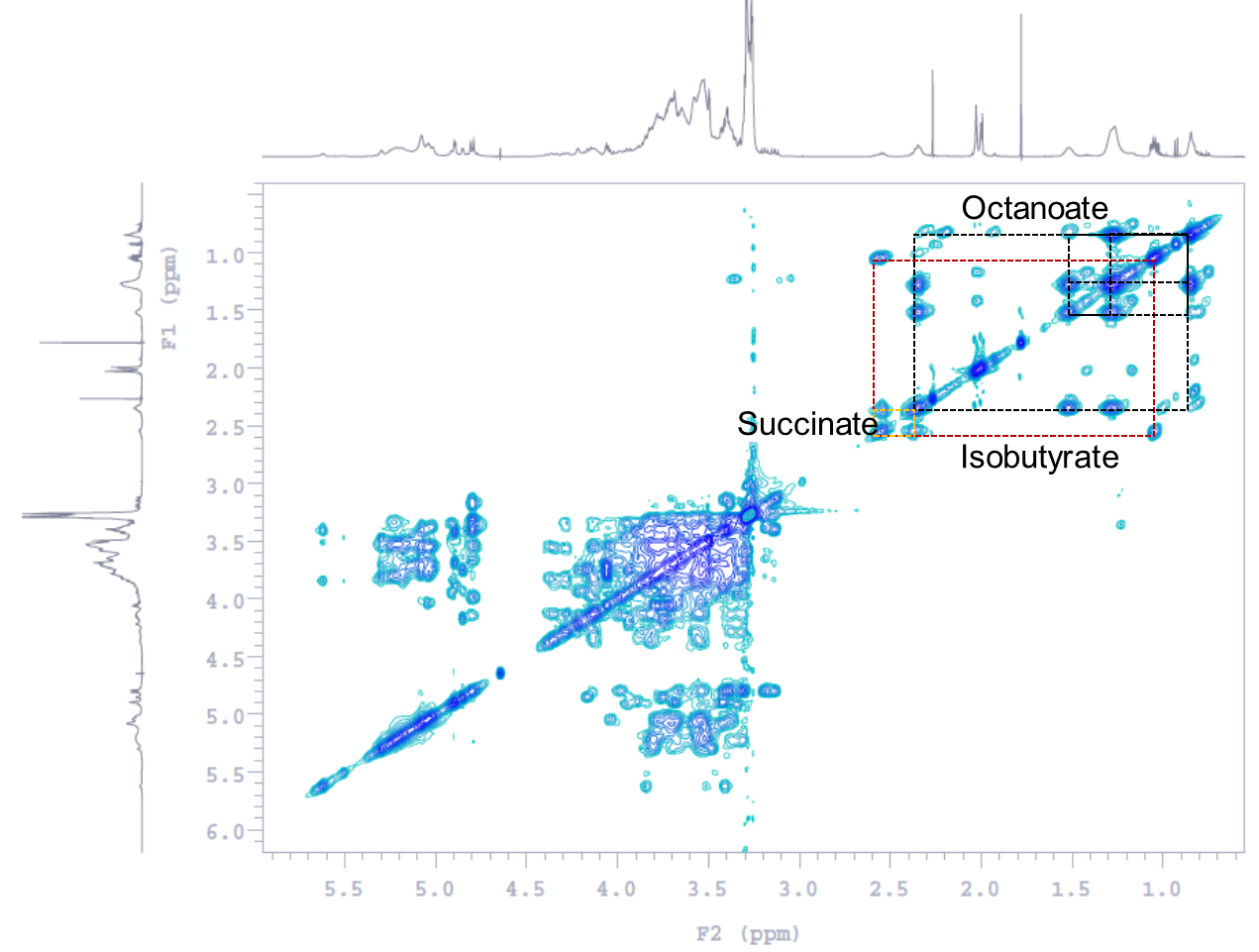


Fig S-2: The Total Correlation Spectroscopy (TOCSY) of native mGLP: Through bond correlation of acyl functionalities present in mGLP.


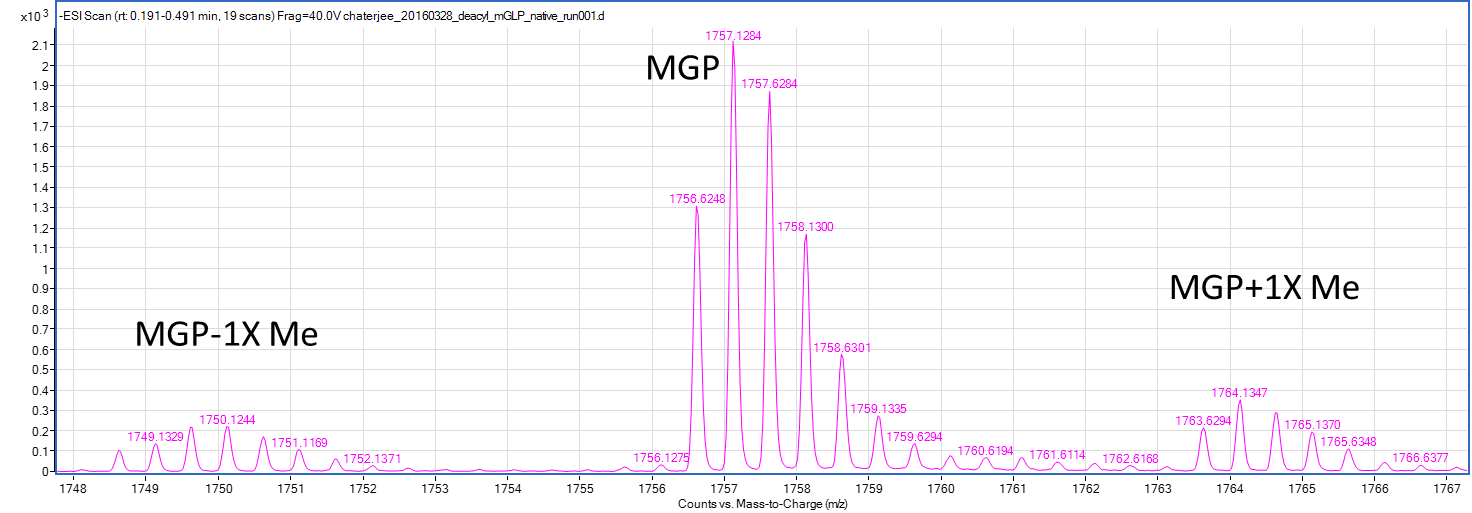


Fig S-3: LC/MS (negative ionization; M-2) of deacyl mGLP (mGP): each ion cluster corresponds to one isoform of mGLP. Additional methyl group (mGP + 1X Me) may indicate biosynthetic chain termination polysaccharide and mGP- 1X Me may represent under-methylated polysaccharide variation of mGLP backbone.


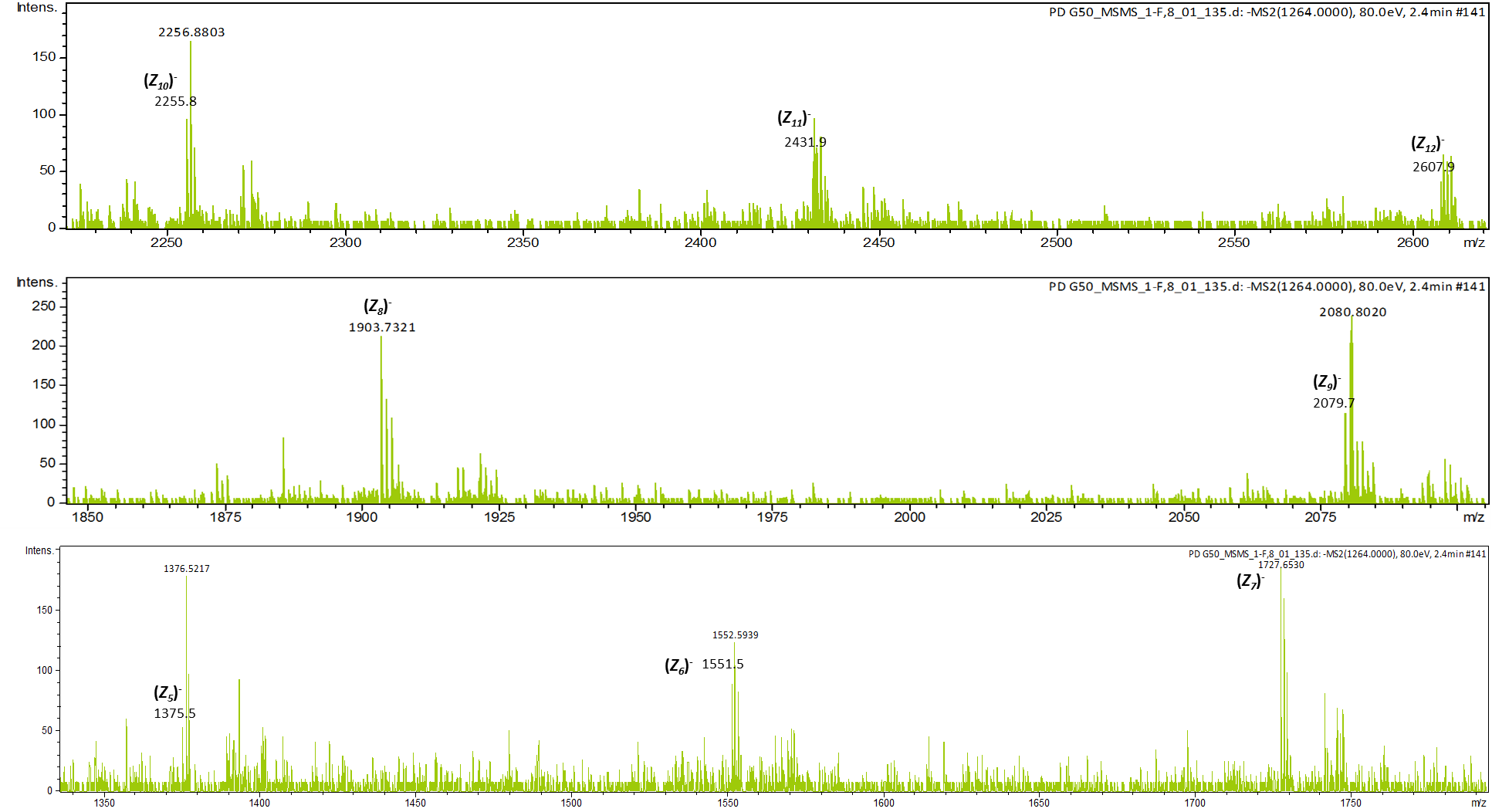


Fig S-4A: MS/MS fragments:m/z 1350-m/z 2620


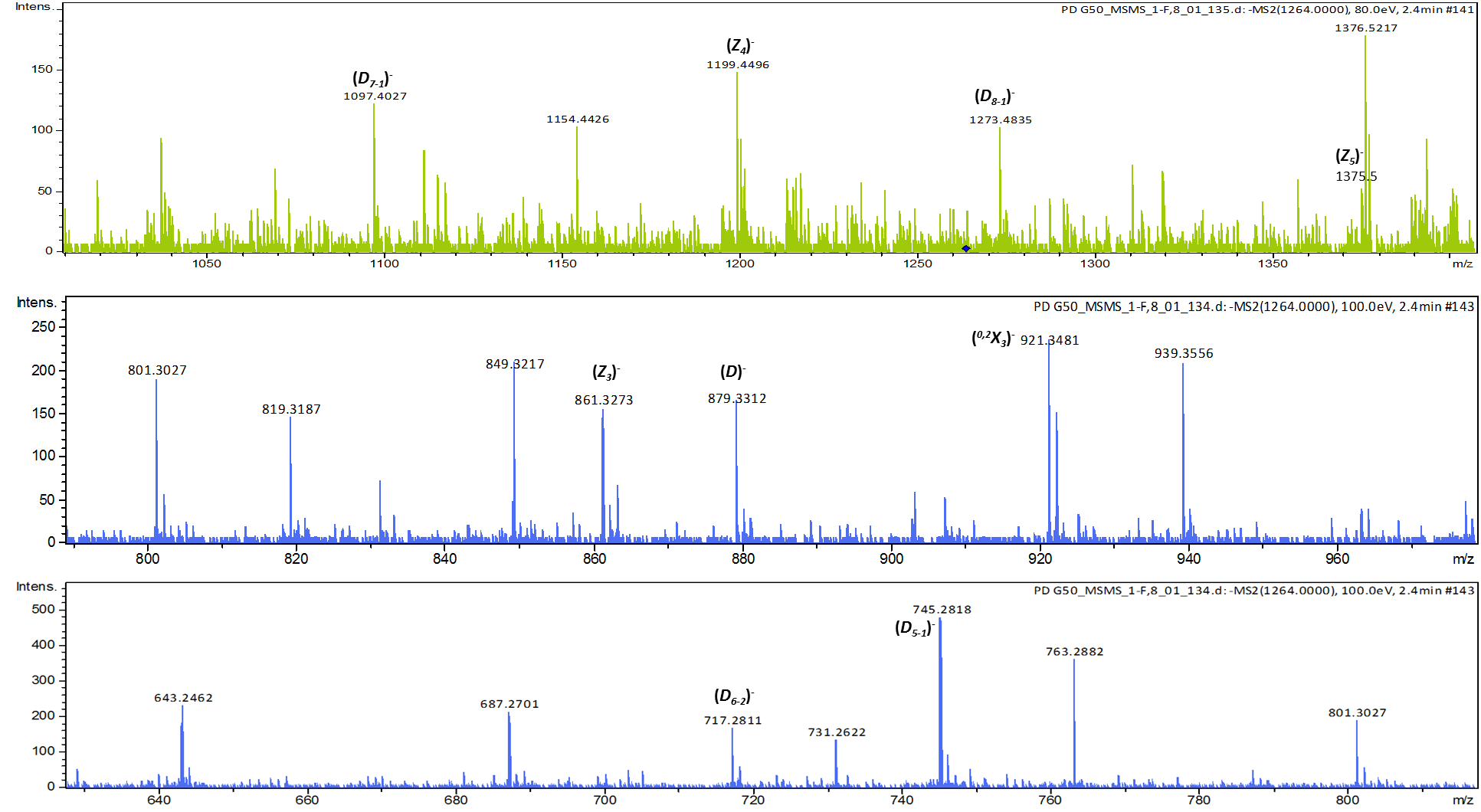


Fig S-4B: MS/MS fragments: m/z 620-m/z1400


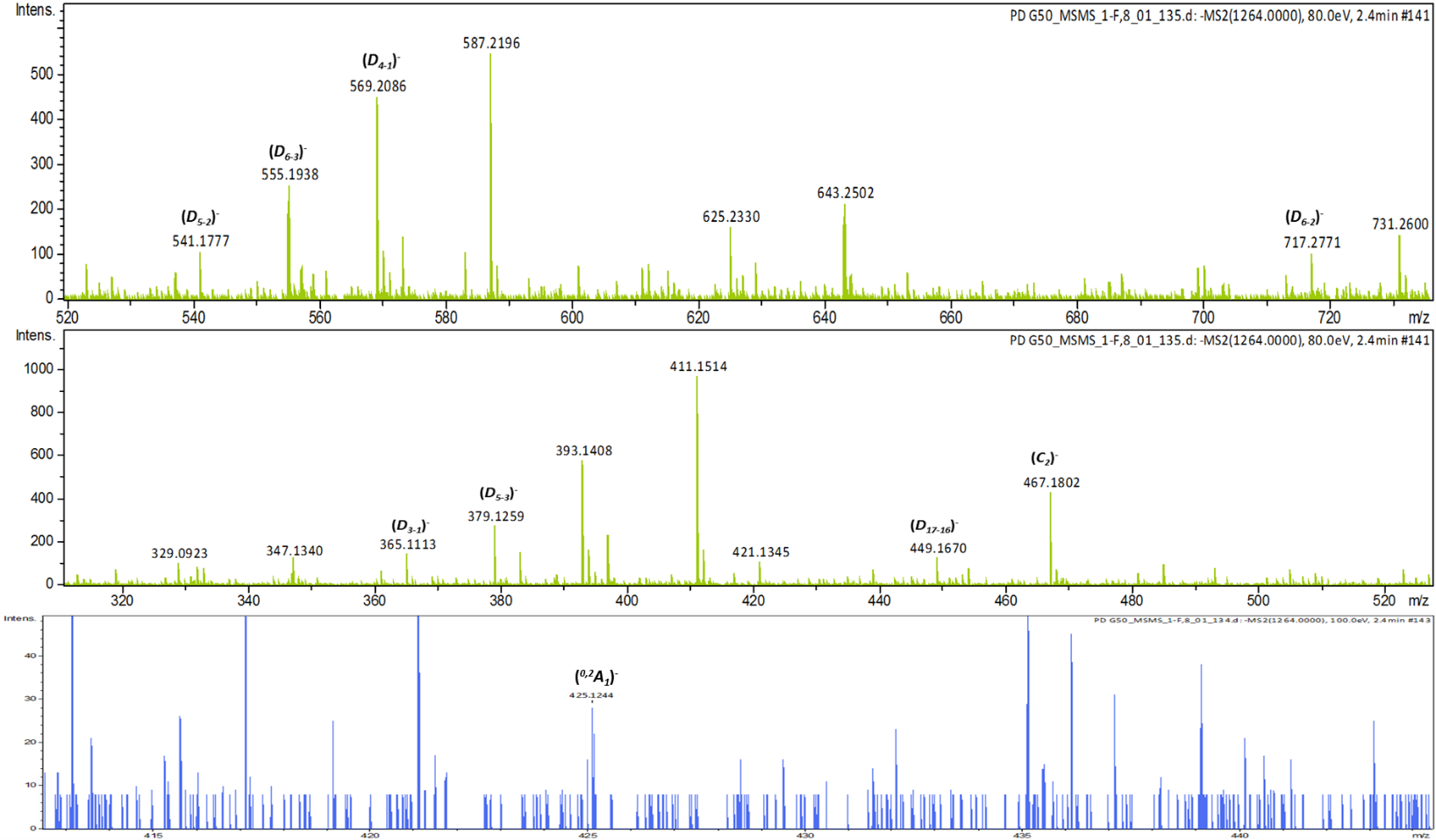


Fig S-4C: MS/MS fragments: m/z 320-720; m/z 425.1


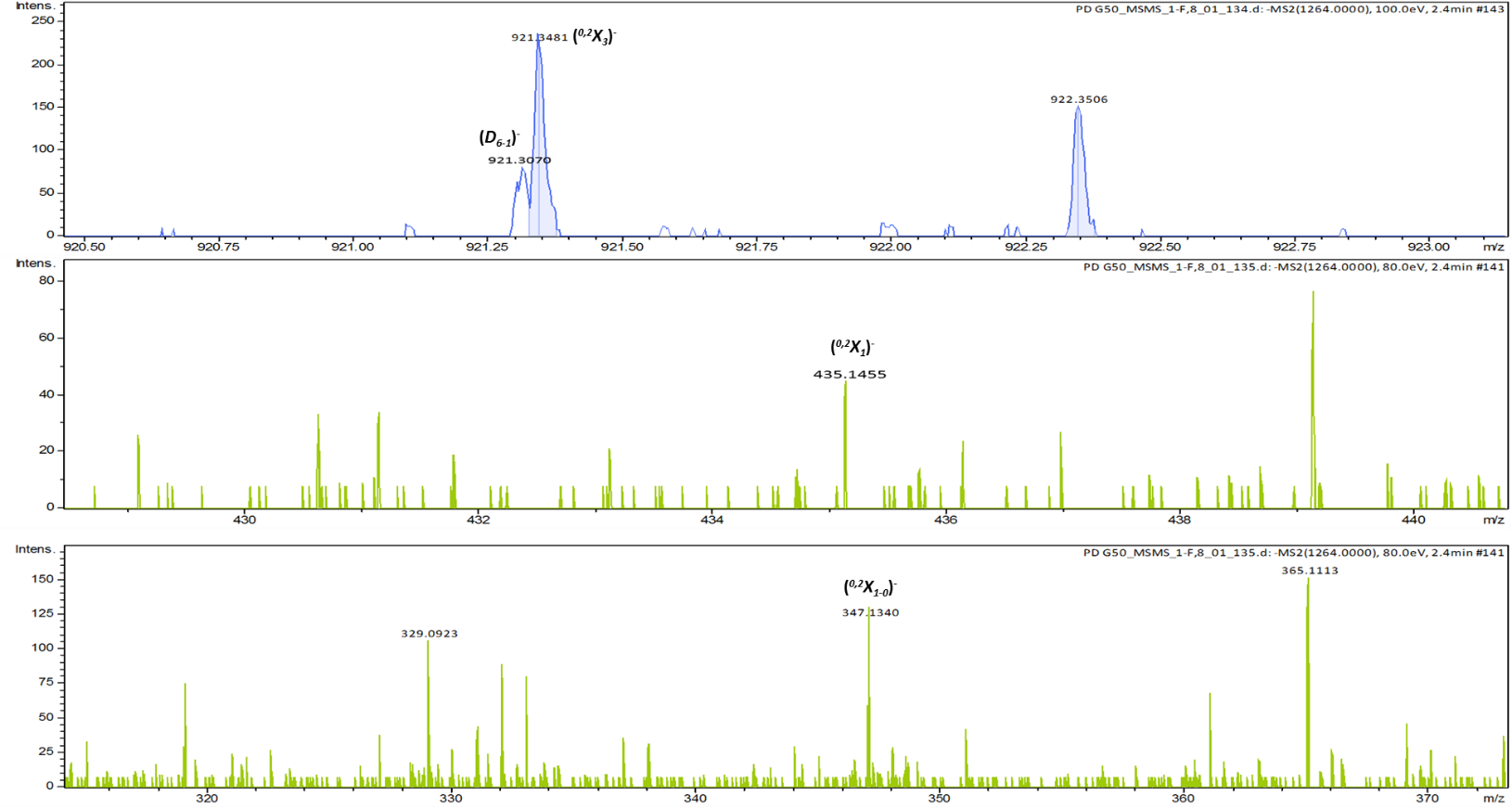


Fig S-4D: MS/MS fragments: m/z 921.30, 921.34; m/z 435.14, m/z 347.13


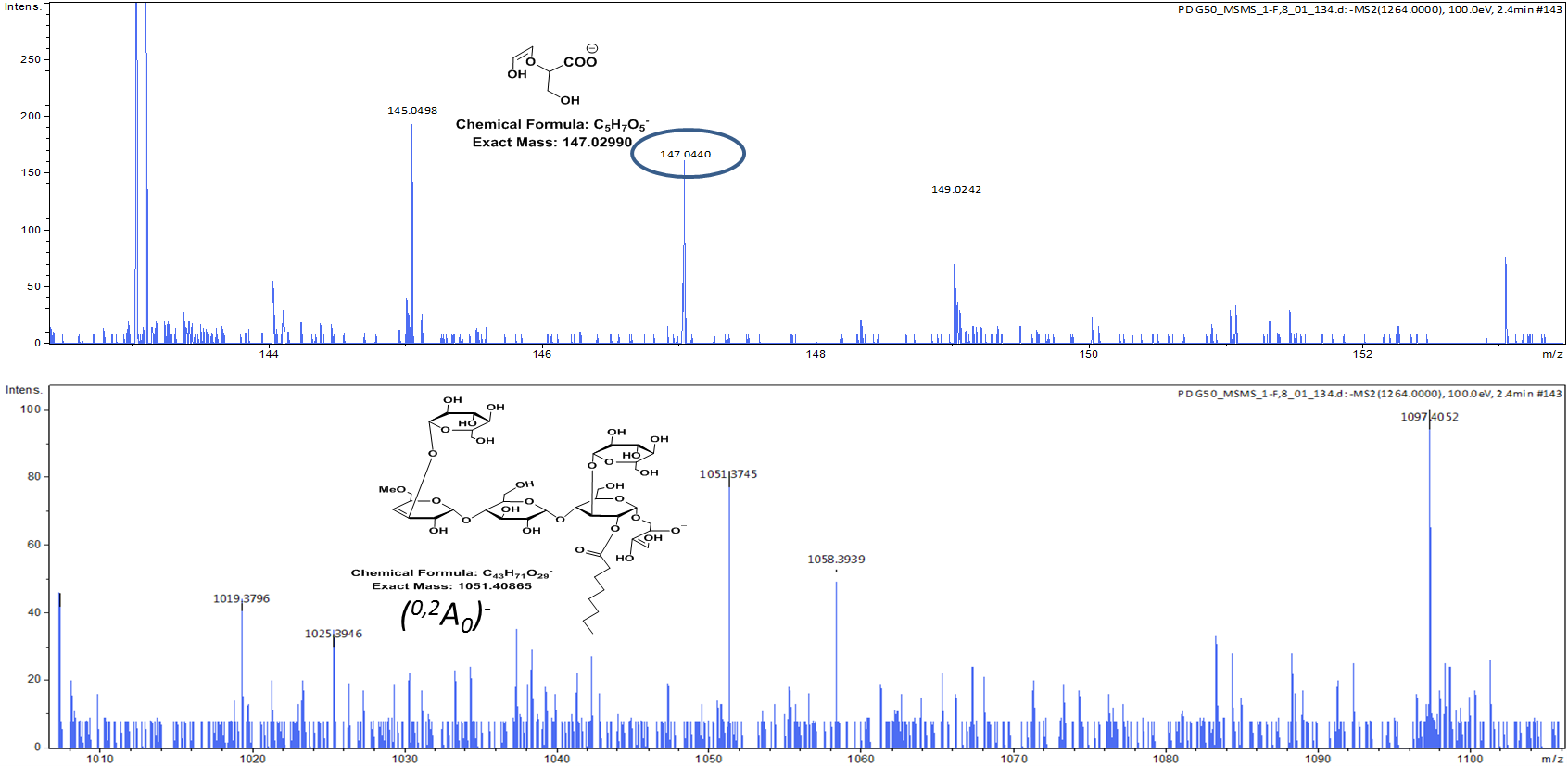


Fig S-4E: MS/MS fragments: m/z 147.0 and 1051.37


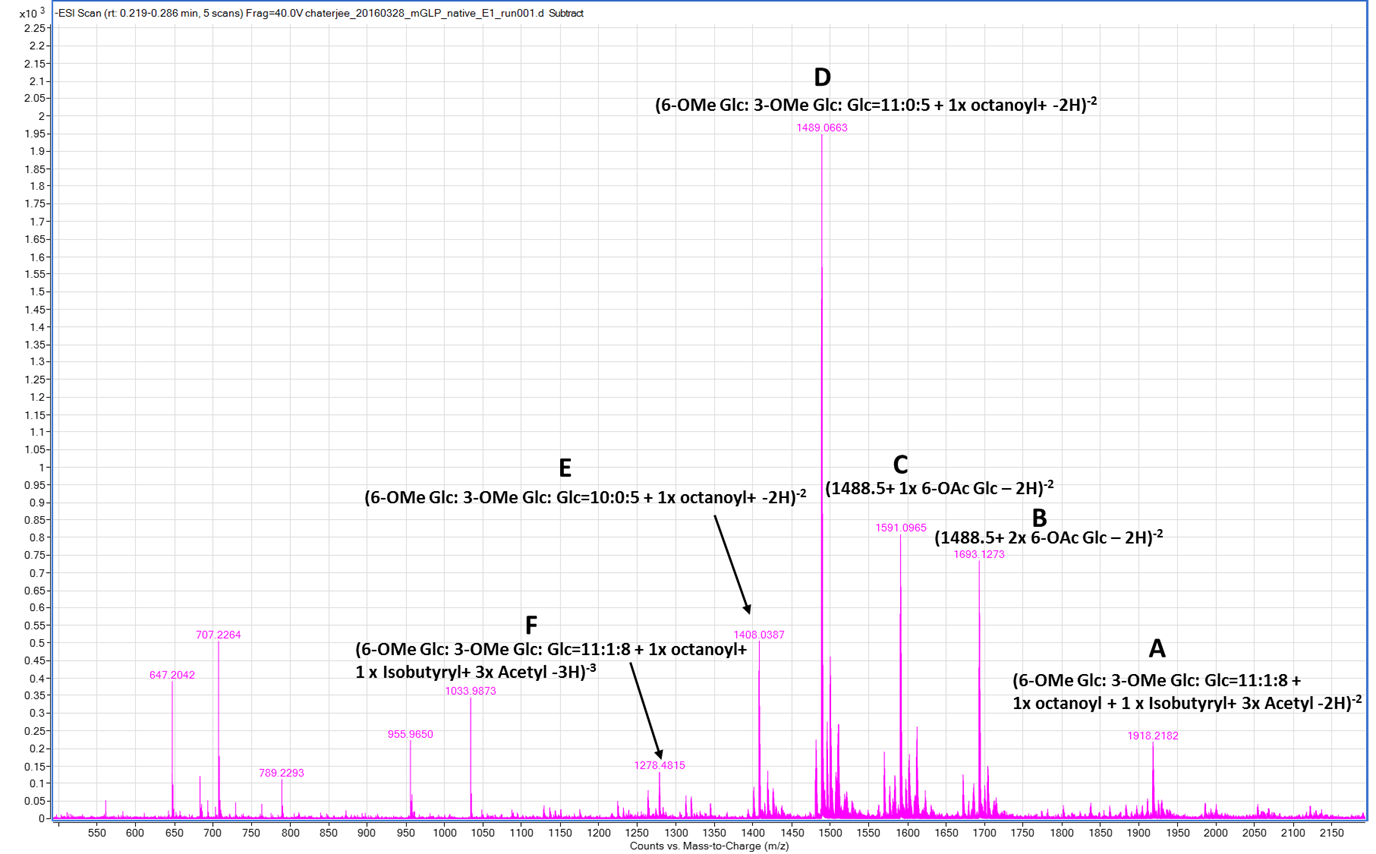


Fig S-5: The LC/MS (m/z 400-3200) of the Crude mixture obtained after α-amylase digestion of mGLP (m/z 1917.7 was the dominant species). A. m/z 1917.7 (M-2H)^-2^: Unreacted mGLP acylform as a result of incomplete digestion. B. m/z 1692.6 (M-2H) ^-2^: Partially digested mGLP resulting from loss of 2 hexoses from non-reducing end. C. m/z 1590.5 (M-2H) ^-2^Partially digested mGLP resulting from loss of 3 hexoses from non-reducing end. D. m/z 1488.5 (M-2H) ^-2^: Major product following enzyme digestion arising from the loss of 4 hexoses from the non-reducing end. E. m/z 1407.5 (M-2H) ^-2^: Minor product following enzyme digestion arising from the loss of 5 hexoses from the non-reducing end. This is arising from the mGLP isoforms which have -1x Me group (as described in Fig 4) in the polysaccharide backbone. D and E shows that the α-amylase activity stops at 6-OMe Glc. F. m/z 1278.1 (M-3H)^-3^: Unreacted mGLP acylform as a result of incomplete digestion.

**Flow chart**


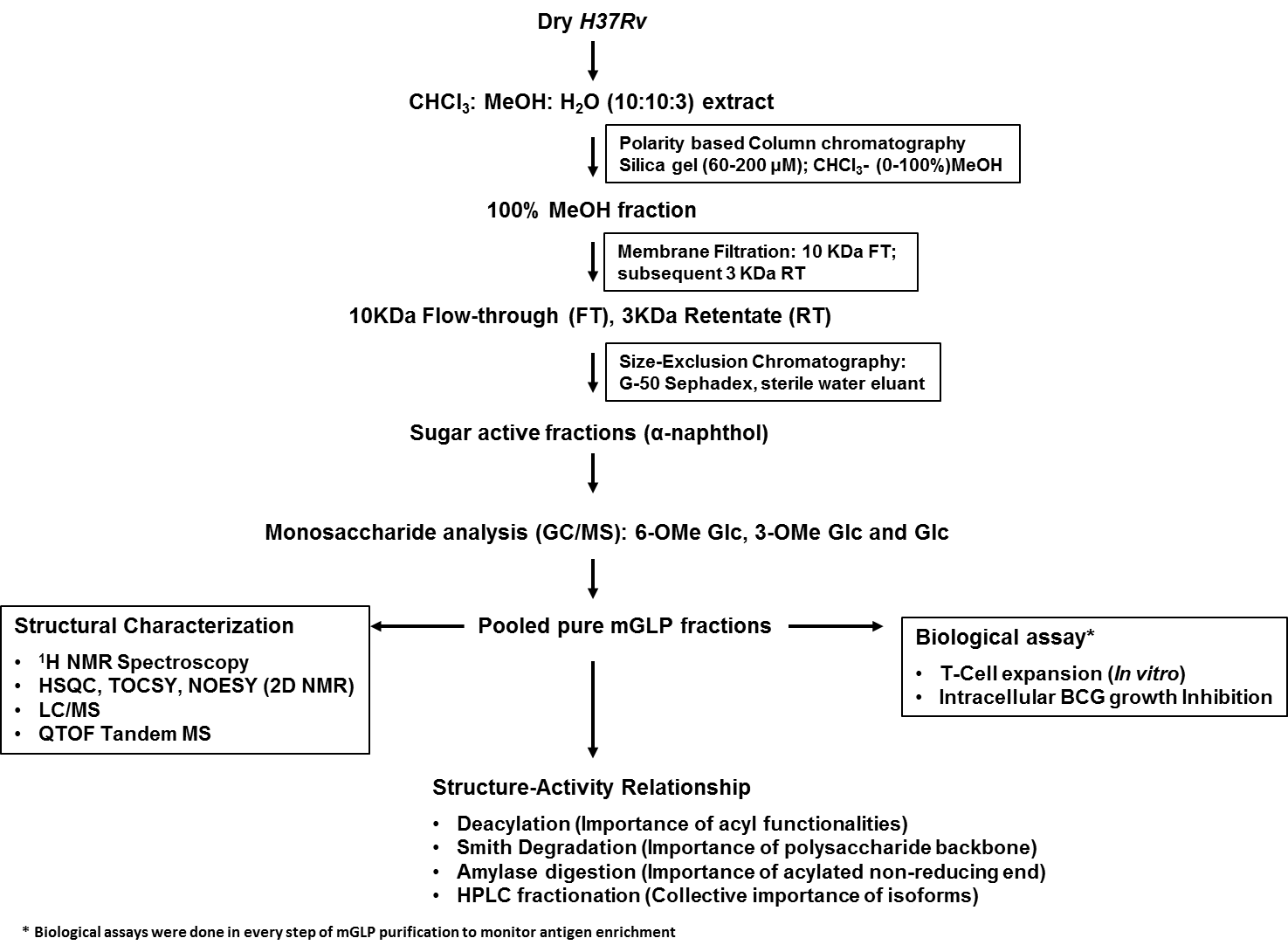


Fig S-6: Overall purification and characterization strategy for Mtb mGLP.
